# *Porphyromonas gingivalis* Gingipain-Dependently Enhances IL-33 Production in Human Gingival Epithelial Cells

**DOI:** 10.1371/journal.pone.0152794

**Published:** 2016-04-08

**Authors:** Hiroyuki Tada, Takashi Matsuyama, Takashi Nishioka, Makoto Hagiwara, Yusuke Kiyoura, Hidetoshi Shimauchi, Kenji Matsushita

**Affiliations:** 1 Department of Oral Disease Research, National Center for Geriatrics and Gerontology, Obu, Aichi, Japan; 2 Division of Oral Microbiology, Tohoku University Graduate School of Dentistry, Sendai, Miyagi, Japan; 3 Division of Oral Diagnosis, Tohoku University Graduate School of Dentistry, Sendai, Miyagi, Japan; 4 Division of Periodontology and Endodontology, Tohoku University Graduate School of Dentistry, Sendai, Miyagi, Japan; 5 Department of Periodontology, Kagoshima University Graduate School of Medical and Dental Sciences, Sakuragaoka, Kagoshima, Japan; 6 Department of Oral Medical Science, Ohu University School of Dentistry, Koriyama, Fukushima, Japan; Tokyo Medical and Dental University, JAPAN

## Abstract

The cytokine IL-33 is constitutively expressed in epithelial cells and it augments Th2 cytokine-mediated inflammatory responses by regulating innate immune cells. We aimed to determine the role of the periodontal pathogen, *Porphyromonas gingivalis*, in the enhanced expression of IL-33 in human gingival epithelial cells. We detected IL-33 in inflamed gingival epithelium from patients with chronic periodontitis, and found that *P*. *gingivalis* increased IL-33 expression in the cytoplasm of human gingival epithelial cells *in vitro*. In contrast, lipopolysaccharide, lipopeptide, and fimbriae derived from *P*. *gingivalis* did not increase IL-33 expression. Specific inhibitors of *P*. *gingivalis* proteases (gingipains) suppressed IL-33 mRNA induction by *P*. *gingivalis* and the *P*. *gingivalis* gingipain-null mutant KDP136 did not induce IL-33 expression. A small interfering RNA for protease-activated receptor-2 (PAR-2) as well as inhibitors of phospholipase C, p38 and NF-κB inhibited the expression of IL-33 induced by *P*. *gingivalis*. These results indicate that the PAR-2/IL-33 axis is promoted by *P*. *gingivalis* infection in human gingival epithelial cells through a gingipain-dependent mechanism.

## Introduction

Epithelial cells play a central role in initiating the innate immune response to pathogens in mucosal tissues, including the oral mucosa. Interleukin (IL)-33 belongs to the IL-1 cytokine family, and it is constitutively expressed in the nuclei of non-immune cells such as fibroblasts, adipocytes, epithelial cells, endothelial cells, and smooth muscle cells, and in some immune cells such as monocytes and dendritic cells [1, 2]. Epithelial cell-derived IL-33 augments Th2 cytokine-mediated inflammation in response to bacterial components [3, 4]. Toll-like receptor ligands and proinflammatory stimuli can up-regulate IL-33 expression [5–8]. Various types of immune cells such as basophils, eosinophils, Th2 cells, mast cells, NKT cells, NK cells, and type 2 innate lymphoid cells (ILC2) express the IL-33 receptor ST2 [9]. Interleukin-33 helps to promote host defense against parasites or bacteria towards Th2 cytokine-associated inflammation [10–14]. In contrast, circumstantial evidence suggests that IL-33 is also involved in the development of inflammatory responses. Interleukin-33 expression is increased in epithelial cells of mucosal lesions arising due to chronic inflammatory diseases such as allergic rhinitis, chronic obstructive lung disease, and chronic colitis [15–18]. Interleukin-33 can control inflammatory responses either positively or negatively. Type 2 innate lymphoid cells produce IL-5 and IL-13 in response to IL-33 and subsequently induce Th2-type inflammation [19–21]. Furthermore, mast cells secrete chemokines in response to IL-33 and subsequently induce neutrophil migration [22]. These activities suggest that IL-33 exerts proinflammatory effects in various chronic inflammatory diseases. However, whether IL-33 is induced in gingival epithelial cells during the development of periodontal disease remains unclear.

*Porphyromonas gingivalis* is a primary pathogen that is involved in chronic periodontitis and it has a diversity of virulence factors that manipulate immune responses, resulting in chronic inflammation and bone loss [23]. This bacterium synthesizes two classes of cysteine proteases; arginine-specific gingipains (RgpA and RgpB) and lysine-specific gingipain (Kgp), which constitutes a major virulence factor [24]. Gingipains are localized in soluble and cell-associated forms, and are secreted as outer membrane blebs [25, 26]. Gingival epithelial cells comprise part of the first line of innate immune responses against *P*. *gingivalis* infection in periodontal tissue. Chronic inflammation results when *P*. *gingivalis* invades gingival epithelial cells [27]. We recently discussed a possible role of IL-33 in the pathogenesis of chronic periodontitis [28]. Although gingival tissues from patients with chronic periodontitis express IL-33 [29], whether or not *P*. *gingivalis* increases IL-33 expression in gingival epithelial cells remains unknown. The present study found that *P*. *gingivalis* upregulates IL-33 expression in human gingival/oral epithelial cells via endogenous gingipain-dependent mechanisms.

## Materials and Methods

### Ethics statement

Gingival tissues were derived from the junctional epithelium of teeth that required extraction from patients (n = 5) with chronic periodontitis at the time of initial examination and from patients (n = 5) without periodontal diseases. The Ethics Committee at Kagoshima University Medical and Dental Hospital reviewed and approved the study protocol (No. 24–143) and all patients provided written, informed consent to the extraction of their teeth and the use of their periodontal tissues for research.

### Reagents

*Porphyromonas gingivalis* fimbriae and PGTP2-RL were a gift from Dr. T. Ogawa (Asahi University). The properties of *P*. *gingivalis* fimbriae [30–31] and PGTP2-RL [32] have been described in detail. The fimbriae were purified from the *P*. *gingivalis* 381 (type I *fim A*) strain prepared by Dr. Ogawa, and LPS containing heterogeneous lipid A structures including tetra- and penta-acylated monophosphorylated lipid A from *P*. *gingivalis* prepared as described by Darveau *et al*. (2004) [33] was purchased from InvivoGen (San Diego, CA). SB203580, cycloheximide and cytochalasin D were obtained from Calbiochem-Novabiochem Co. (La Jolla, CA). U-73122, GF109203X, ammonium pyrrolidine dithiocarbamate (PDTC) and dimethyl sulfoxide (DMSO) were sourced from Sigma-Aldrich (St. Louis, MO). Phe-Pro-Arg-chloromethyl ketone (FPR-cmk) was obtained from Enzo Life Sciences (Farmingdale, NY) and KYT-36 was sourced from the Peptide Institute (Osaka, Japan).

### Culture of *P*. *gingivalis* and preparation of whole cell preparations

*Porphyromonas gingivalis* W83, ATCC 33277, KDP131 (Δ*rgpA*), KDP132 (Δ*rgpB*), KDP129 (Δ*kgp*), KDP133 (Δ*rgpA* Δ*rgpB*) and KDP 136 (Δ*kgp* Δ*rgpA* Δ*rgpB*) [34] were cultured anaerobically (10% CO_2_, 10% H_2_, and 80% N_2_) at 37°C for 60 h in enriched tryptic soy broth containing 1% tryptone (Difco, Detroit, MI), 3% tryptic soy (Becton Dickinson Co., Franklin Lakes, NJ), 2.5% yeast extract (Becton Dickinson), 0.1% _L_-cysteine (Wako Pure Chemical Industries, Osaka, Japan), 5 μg/mL of hemin, and 5 μg/mL of menadione. After three washes with PBS, whole bacterial cells at the log-phase of growth were resuspended in PBS, lyophilized by centrifugation *in vacuo* and then resuspended in distilled water. Viability monitored by counting colony-forming units (CFU) in blood tryptic soy agar, was 4 × 10^4^ CFU/50 μg of lyophilized whole bacterial cells. Enzyme activities in these cells were blocked by a 15-min incubation with the Rgps inhibitor FPR-cmk and/or the Kgp inhibitor KYT-36 at room temperature (RT) before using the bacterial cells to stimulate human gingival epithelial Ca9-22 cells.

### Cell and cell lines

The human gingival epithelial cell line Ca9-22 derived from squamous cell carcinoma was obtained from the Japanese Collection of Research Bioresources Cell Bank (Hokkaido, Japan). Ca9-22 cells were cultured in E-MEM (Wako Pure Chemical Industries) supplemented with 10% heat-inactivated fetal bovine serum (FBS) (HyClone, Logan, UT) and 1% antibiotic-antimycotic mixture (Life Technologies, Grand Island, NY). Human oral epithelial cells (ScienCell Research Laboratories, Carlsbad, CA) were cultured in keratinocyte serum-free medium (Life Technologies). The ability of primary oral epithelial cells to adhere to culture plates slightly decreased after stimulation with *P*. *gingivalis*. Therefore, we analyzed the Ca9-22 cell line in this study, because it adhered more tightly to culture plates than primary oral epithelial cells after *P*. *gingivalis* stimulation. Cells were incubated in medium containing 5% FBS unless otherwise indicated.

### IL-33 detection by immunohistochemistry

Gingival tissues from the junctional epithelium of patients with chronic periodontitis (n = 5) were obtained from teeth that required extraction at the time of initial examination. The criteria for chronic periodontitis comprised: probing pocket depth ≥ 5.0 mm, bleeding upon probing, clinical attachment loss ≥ 5.0 mm and radiographically confirmed extensive bone loss. The criteria for periodontally healthy individuals comprised: probing pocket depth < 3.0 mm, no bleeding upon probing, clinical attachment loss < 3.0 mm, and scarcely bone loss confirmed radiographically. Frozen gingival tissues embedded in OCT compound (Sakura Finetek, Tokyo, Japan) were sliced into 4-μm-thick sections. Endogenous peroxidase was blocked by incubation with 0.5% H_2_O_2_ for 5 min. The sections were then incubated with 2 μg/ml of rabbit anti-human IL-33 mAb (EPITOMICS, Burlingame, CA) or an isotype-matched control for 1 h at RT and with horseradish peroxidase (HRP)-conjugated goat anti-rabbit IgG (Cell Signaling Technology, Beverly, MA). The sections were immunostained with 3,3’-diaminobenzidine (DAB) substrate (DakoCytomation, Carpinteria, CA), counterstained with Mayer’s hematoxylin and observed by microscopy.

### Reverse transcription and real-time polymerase chain reaction (RT-qPCR)

Total cellular RNA was extracted using RNeasy^®^ (QIAGEN Inc., Valencia, CA) according to the manufacturer’s instructions and treated with DNase (RNase-free DNase set, QIAGEN). Total RNA (1 μg) was reverse-transcribed using Transcriptor First Strand cDNA Synthesis Kits^®^ (Roche Diagnostic Co., Indianapolis, IN) and a 1:10 volume of cDNA was theoretically converted from 100 ng of total RNA. Primers for PCR were designed using LightCycler probe design software^®^ (Roche Diagnostics GmbH, Mannheim, Germany), and comprised: IL-33, 5’-TTAAATAGGGTATTGGTAAAGAAACGG-3’ and 5’- AGTGTTTGAGCCTATCGTTTG-3’; IL-25, 5’-CCTACAGACAGGCTCCC-3’ and 5’-ACACACACAAGCTAAGGAAACA-3’; TSLP, 5’-TCTCAAATCTAGTTAGACAATTTGCAC-3’ and 5’-CATCCTGAAGCTGCCTATC-3’; PAR-1, 5’-TCGTCACTGCAGCATTT-3’ and 5’-AGATGGCCAGACAAGTGAA-3’; PAR-2, 5’-CTTTGCCGAAGTGTCCG-3’ and 5’-CCTACTGTGCAATTCCCA-3’; PAR-3, 5’-TTCCATTTGCCTTATTGCTACT-3’ and 5’-TGAGGGACCCTACAAAGATAGA-3’; PAR-4, 5’-GCTGTACTGGGTCGAAC-3’ and 5’-CCTGGCCTCTCCTTATCT-3’; GAPDH, 5’-TGAACCATGAGAAGTATGACAACA-3’ and 5’-TCTTCTGGGTGGCAGTG-3’. The amplification profile was 40 cycles at 94°C for 60 s, 55°C for 30 s, and 72°C for 30 s. PCR amplification proceeded using an Applied Biosystems 7300 Real-time PCR System^®^ (Life Technologies). The relative induction of IL-33 mRNA expression was determined after normalization using GAPDH as the reference gene and values are shown vs. control, which was converted to 1. For conventional PCR, The amplification profile was 30 cycles using the same program. Amplified samples were resolved on 2.0% agarose gels, stained with ethidium bromide and photographed under UV light.

### Western blotting

Confluent cell monolayers were harvested using cell lysis buffer^®^ (Cell Signaling Technology) according to the manufacturer’s instructions. Nuclear and cytoplasmic extracts were separated using NE-PER nuclear and cytoplasmic extraction reagents (Pierce Biotechnology, Rockford, IL), respectively. Whole cell lysates, as well as nuclear and cytoplasmic fractions were separated by SDS-PAGE, and transferred to polyvinylidene difluoride membranes (ATTO, Tokyo, Japan) using a semidry transblot system (Bio-Rad Laboratories, Hercules, CA). Non-specific binding on the blots was blocked with 0.5% (w/v) non-fat dried milk and 0.1% (v/v) Tween 20 in PBS overnight at 4°C, followed by incubation for 1 h at RT with rabbit anti-human IL-33 mAb (EPITOMICS), mouse anti-human GAPDH mAb (MBL, Tokyo, Japan), rabbit anti-human p38 (Cell Signaling Technology), or with p38 polyclonal antibodies (Cell Signaling Technology) at 1:1,000 for 1 h at RT. Blots were incubated with HRP-conjugated goat anti-rabbit IgG (Cell Signaling Technology) at 1:2,000 for 1 h at RT and then soaked in Immunostar^®^ (Wako Pure Chemical Industries). Chemiluminescent signals were detected using an LAS-4000IR luminescent image analyzer (Fujifilm, Tokyo, Japan). The intensity of expression was quantified by densitometry using ImageJ software.

### IL-33 detection by immunofluorescence assay

A suspension of Ca9-22 cells (3 × 10^5^/ml) was seeded overnight onto ibiTreat μ-Slide VI ^0.4^ (ibidi GmbH, Martinsried, Germany) and stimulated with 50 μg/ml of whole *P*. *gingivalis* cells at MOI of 0.13 for 4 d to assess intracellular IL-33 expression. The cells were fixed with 4% paraformaldehyde for 15 min, permeabilized with PBS containing 0.1% saponin for 10 min and then incubated in PBST containing 1% (w/v) BSA for 30 min to block non-specific binding. The cells were incubated for 1 h at 4°C with 0.5 μg/ml of phycoerythrin conjugated-rat anti-human IL-33 mAb (clone, 390412; R&D Systems, Minneapolis, MN). Thereafter, the cells were incubated with DAPI for 1 min to identify nuclei, mounted on slides using mounting medium, and assessed using fluorescence microscopy (BZ-9000, Keyence, Tokyo, Japan).

### RNA interference

To inhibit human protease-activated receptor (PAR)-2 or β-arrestin-1 expression, Ca9-22 cells were transfected with Ambion Silencer^®^ Select Validated small interfering RNA (siRNA) for PAR-2, β-arrestin-1, or negative control (Life Technologies). Cells were seeded on a 24-well plates, and then transfected with 100 pmol of siRNA in 0.5 μl of Lipofectamine^®^ 2000 (Life Technologies) according to the manufacturer’s protocol. The transfected cells were stimulated 12 h later with whole *P*. *gingivalis* W83 cells for 48 h and assayed as described.

### NF-κB luciferase assay

The pNFκB-*Metridia* luciferase reporter plasmid contains an NF-κB promoter element upstream of the secreted *Metridia* luciferase (MetLuc) gene (Clontech Laboratories, Mountain View, CA). The p*Metridia* luciferase reporter plasmid contains several cloning sites that allow the insertion of promoter and/or enhancer elements upstream of the secreted MetLuc gene (Clontech Laboratories), which serves as an internal standard of transfection. Transient transfection was achieved using Lipofectamine^®^ LTX (Life Technologies) according to the manufacturer’s instructions. Briefly, sub-confluent cells in 24-well plates were transiently transfected with 0.5 μg of the reporter construct and then stimulated 24 h later with whole *P*. *gingivalis* cells for 48 h in E-MEM containing 5% FBS. Secreted *Metridia* luciferase activity in culture supernatants was analyzed using Ready-To-Glow™ Secreted Luciferase Reporter Systems (Clontech Laboratories) and a SpectraMax M5 luminometer (Molecular Devices, Menlo Park, CA).

### Measurement of IL-33 production by ELISA

Cells were stimulated with whole *P*. *gingivalis* cells for 48 h. Levels of IL-33 in culture supernatants were measured using IL-33 ELISA kits (eBioscience, San Diego, CA) according to the manufacturer’s instructions. The absorbance of IL-33 was determined using the SoftMax^®^ Pro data analysis program (Molecular Devices).

### Enzyme activity assay

The amidolytic activities of gingipains secreted from whole *P*. *gingivalis* cells were assayed at 37°C using 0.5 mM N-α-benzoyl-_L_-arginine-*p*-nitroanilide (BA-pNA) for Rgps and 0.5 mM Z-His-Glu-Lys-4-methylcoumaryl-7-amide (HEK-MCA) for Kgp in 1.0 ml of 0.2 M Tris-HCl, 0.1 M NaCl, 5 mM CaCl_2_, and 10 mM cysteine (pH 7.6). The absorbance of *p*-nitroaniline or 7-amino-4-methylcoumarin released from the substrate was measured at 405 or 370 nm, respectively.

### Statistical analysis

All experiments were repeated at least three times in triplicate to test the reproducibility of the results. Experimental data are representative results and are shown as means ± SD. The statistical significance of differences between control and experimental findings was evaluated by post-test after ANOVA. *p*-values < 0.05 were considered significant.

## Results

### Expression of IL-33 in human periodontal tissues

**Fig 1:**
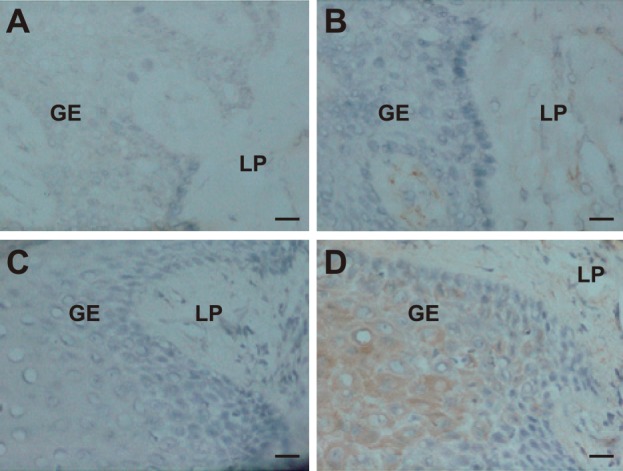
Production of IL-33 is increased in chronic periodontitis. Cryosections of normal periodontal tissues from healthy donors (A and B) and inflamed periodontal tissues from patients with chronic periodontitis (C and D) were stained with rabbit anti-human IL-33 mAb (B and D) or rabbit anti-human IgG isotype control (A and C) and HRP-conjugated anti-rabbit IgG and then visualized with DAB. Sections were counterstained with Mayer’s hematoxylin. GE, gingival epithelium; LP, lamina propria. Bar = 3 μm. Data are representative of five experiments.

We initially analyzed IL-33 expression in gingival tissues from patients with chronic periodontitis by immunohistochemical staining to determine whether inflamed gingival epithelium expresses IL-33. Interleukin-33 was expressed in the cytoplasm of inflamed gingival epithelium from patients with chronic periodontitis (Fig 1D), but scarcely in that from healthy individuals (Fig 1B). In contrast, cytokines were weakly expressed in the lamina propria of gingival tissues (Fig 1B and 1D). The results from gingival tissues collected from at least four other patients with chronic periodontitis were similar (data not shown). An isotype-matched control antibody did not react with these tissues (Fig 1A and 1C). These findings suggest that gingival epithelium is a major source of IL-33 after infection with periodontal pathogens.

### *P*. *gingivalis* increases IL-33 mRNA expression in human gingival epithelial cells

**Fig 2:**
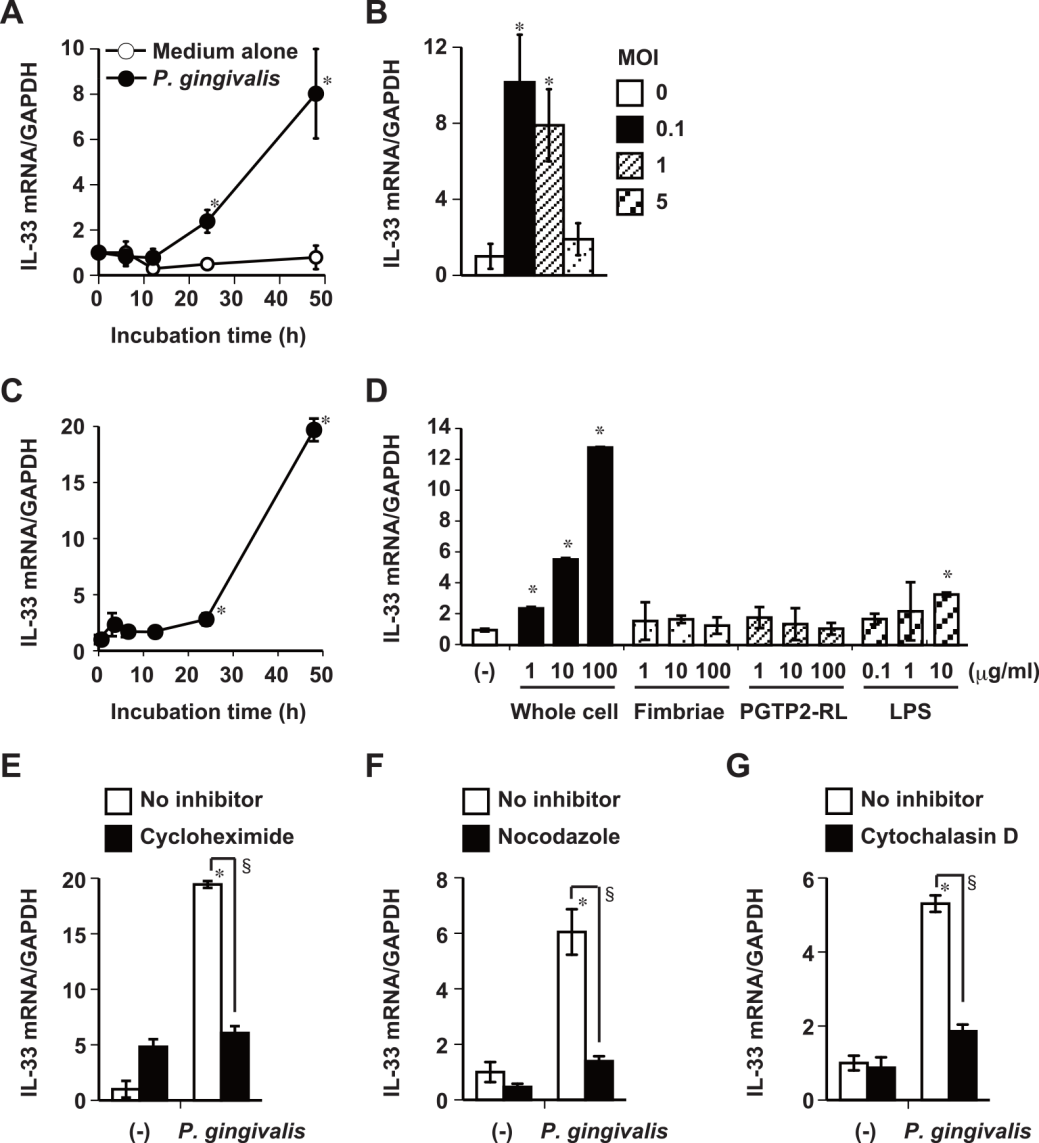
*P*. *gingivalis* increases IL-33 mRNA expression in gingival epithelial cells. Ca9-22 cells were infected with fresh *P*. *gingivalis* W83 cultures at MOI of 0.5 for the indicated periods (A) or indicated MOI for 48 h (B). Ca9-22 cells were stimulated with 50 μg/ml (MOI of 0.1) of whole *P*. *gingivalis* W83 cells for the indicated periods (C) and indicated amounts of whole *P*. *gingivalis* W83 cells, fimbriae, PGTP2-RL, and LPS derived from *P*. *gingivalis* for 48 h (D). Cells were incubated with 10 μg/ml cycloheximide for 45 min (E), 1 μg/ml nocodazole for 1 h (F) or 0.5 μM cytochalasin D for 30 min (G) and then stimulated for 48 h with 50 μg/ml of *P*. *gingivalis* W83 whole cells with the respective inhibitors. Total cellular RNA was then extracted at the indicated times, and IL-33 transcripts were analyzed by RT-qPCR. Data are representative of three independent experiments, and are shown as means ± SD of triplicate assays. Statistically significant differences are indicated. *, *P*<0.05 compared with untreated control; §, *P*<0.05 compared with *P*. *gingivalis* alone.

The suspected major pathogen associated with chronic periodontitis is *P*. *gingivalis* [23]. We therefore analyzed the effects of infection with viable *P*. *gingivalis* upon IL-33 mRNA expression in human gingival epithelial (Ca9-22) cells *in vitro*. The expression of IL-33 mRNA in Ca9-22 cells significantly increased from 24 to 48 h after infection with *P*. *gingivalis* W83 (Fig 2A and 2B) and after stimulation with lyophilized whole cells of *P*. *gingivalis* (Fig 2C). Therefore, IL-33 mRNA expression was mainly analyzed at 48 h after stimulation. We then examined whether IL-33 mRNA can be induced by bacterial cell wall components: fimbriae (TLR2 ligand) [31] and LPS (TLR2 and 4 ligand) [33] prepared from *P*. *gingivalis*, and synthetic *P*. *gingivalis*-type lipopeptide PGTP2-RL (TLR2/1 ligand) [35]. Fig 2D shows that IL-33 mRNA expression was significantly and dose-dependently enhanced after stimulation with > 1 μg/ml *P*. *gingivalis* W83, whereas fimbriae and PGTP-2-RL from *P*. *gingivalis* and the LPS preparations were inactive and slightly active, respectively. In addition to IL-33, IL-25, and TSLP are also produced by epithelial cells at mucosal surfaces, and they can induce the activation of Th2 cytokine-mediated immune responses [3]. Infection with *P*. *gingivalis* W83 (S1A and S1B Fig) or stimulation with whole *P*. *gingivalis* cells (S1C Fig) enhanced IL-25 and TSLP mRNA expression. In the presence of a protein synthesis inhibitor cycloheximide, *P*. *gingivalis* whole cells did not induce the expression of IL-33 mRNA in epithelial cells incubated with the protein synthesis inhibitor, cycloheximide (Fig 2E), indicating that the induction of IL-33 mRNA by *P*. *gingivalis* requires *de novo* protein synthesis. Furthermore, incubating the cells with nocodazole, a microtubule inhibitor, or with cytochalasin D, a particle internalization inhibitor, blocked IL-33 mRNA induction by *P*. *gingivalis*, suggesting that internalization of the bacterium is required for IL-33 mRNA induction (Fig 2F and 2G).

### *P*. *gingivalis* increases IL-33 protein expression

**Fig 3:**
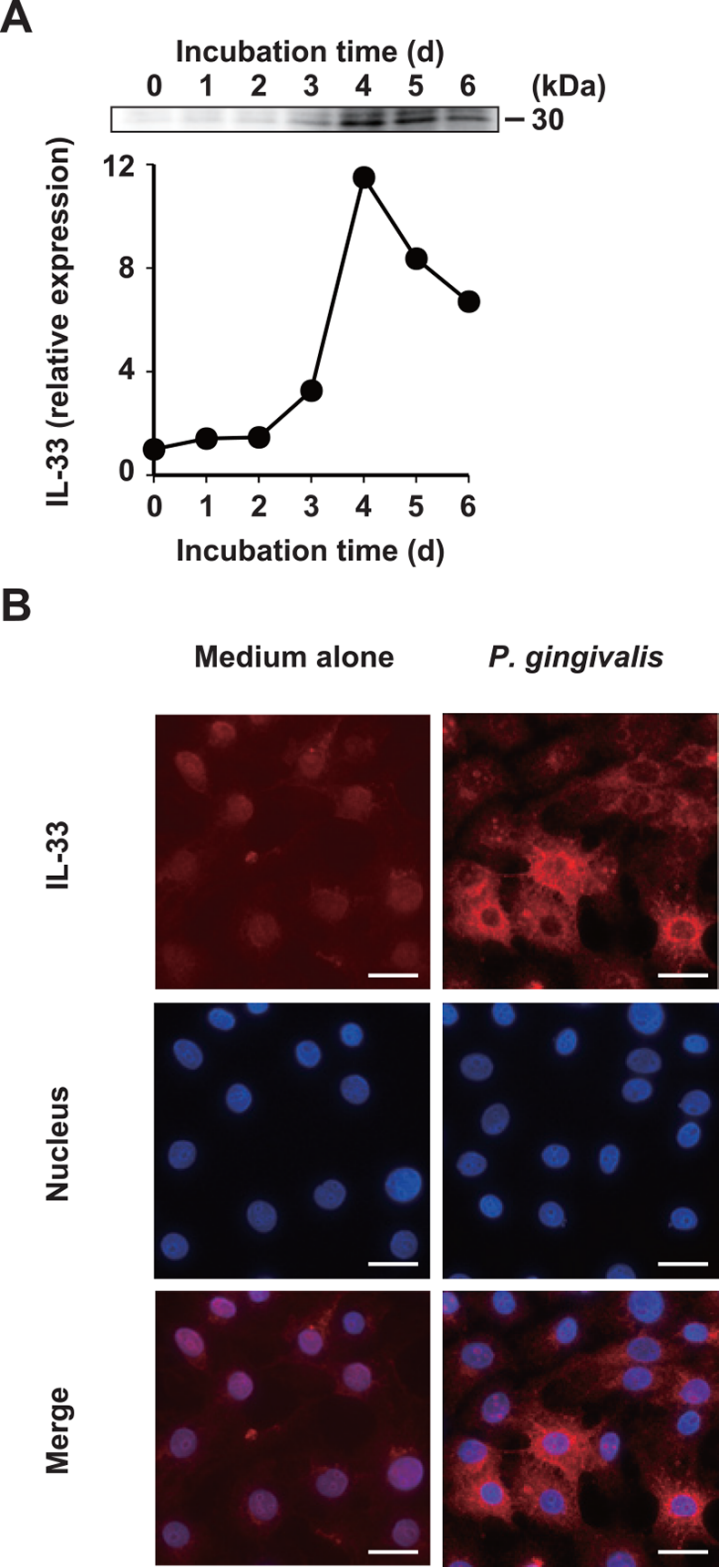
*P*. *gingivalis* increases IL-33 protein expression in gingival epithelial cells. (A) Ca9-22 cells were stimulated with 50 μg/ml of whole *P*. *gingivalis* W83 cells for the indicated periods. Cell lysates were analyzed by Western blotting with an anti-human IL-33 mAb. Expression levels of IL-33 were quantified by densitometry using ImageJ software and normalized to medium alone. (B) Ca9-22 cells were stimulated with 50 μg/ml of whole *P*. *gingivalis* W83 cells for 4 d, and intracellular IL-33 protein was stained with PE-conjugated anti-human IL-33 mAb. Nuclei were stained with DAPI. Bar = 25 μm. Data are representative of three independent experiments.

Stimulating human epithelial cells with extracellular ATP or applying mechanical stress without cell death results in the production of IL-33 [36, 37]. We examined whether *P*. *gingivalis* can induce IL-33 in Ca9-22 cells using Western blotting. Interleukin-33 was constitutively localized in the nuclei of resting Ca9-22 cells (S2 Fig) and its expression was increased 11.5-fold, reaching a peak at four days after *P*. *gingivalis* stimulation (Fig 3A). However, IL-33 levels in the supernatants from the same cultures were quite low (8–25 pg/ml) according to the ELISA findings (data not shown). Therefore, we examined the immunocytochemical localization of IL-33 protein in Ca9-22 cells after stimulation with *P*. *gingivalis*. Interleukin-33 accumulated in the cytoplasm of Ca9-22 cells after *P*. *gingivalis* stimulation for 4 d regardless of its constitutive localization in the nuclei of the resting cells (Fig 3B and S2 Fig).

### Gingipains participate in increased expression of IL-33 mRNA induced by *P*. *gingivalis*

**Fig 4:**
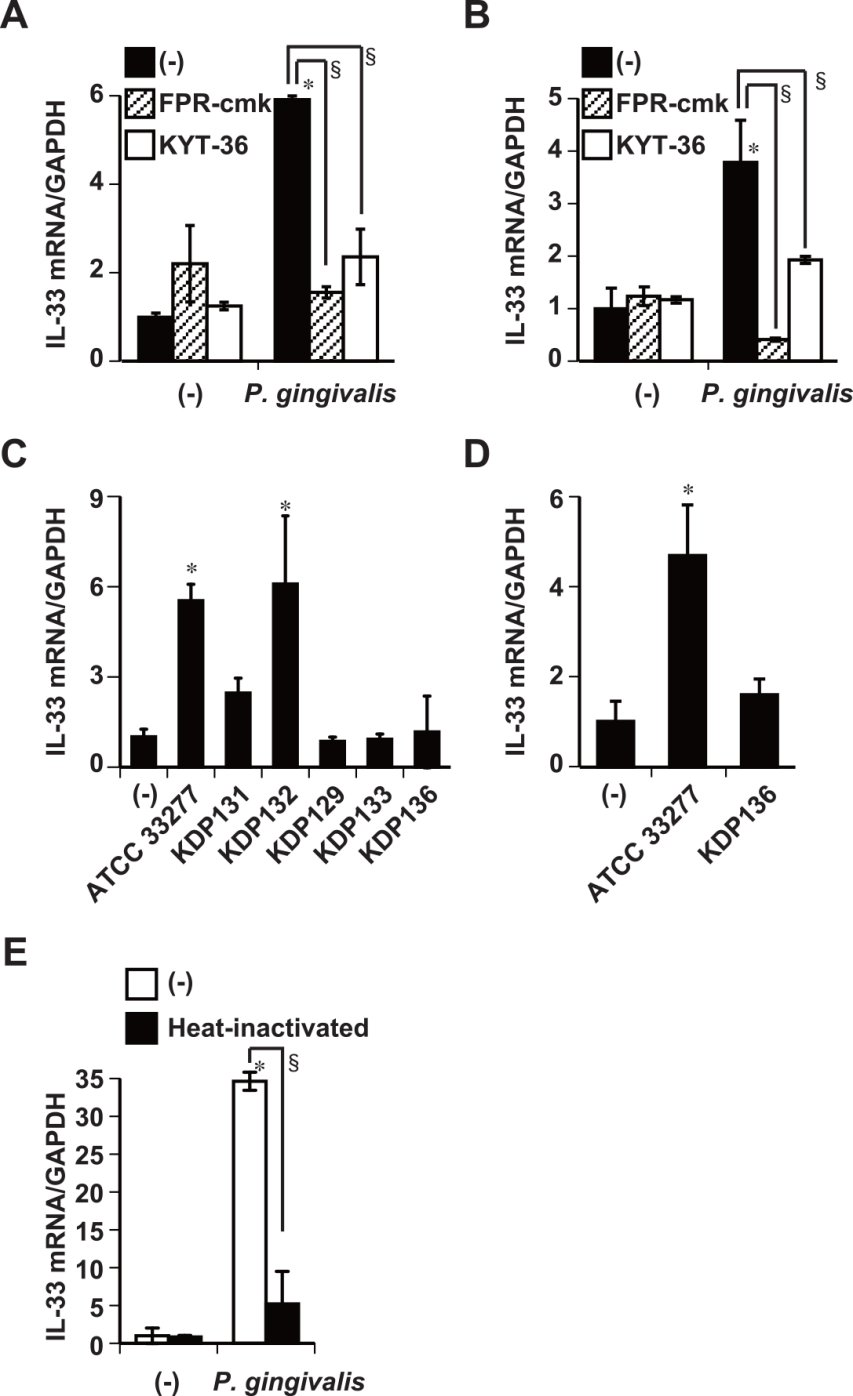
Participation of gingipains in *P*. *gingivalis*-induced IL-33 mRNA expression in gingival/oral epithelial cells. Whole *P*. *gingivalis* W83 cells (50 μg/ml) were incubated with 0.3 μM FPR-cmk (Rgp inhibitor) or 0.3 μM KYT-36 (Kgp inhibitor) for 15 min at 37°C and then used to stimulate Ca9-22 (A) or primary oral epithelial (B) cells for 48 h. (C) Ca9-22 cells stimulated with 50 μg/ml of whole cells of *P*. *gingivalis* ATCC 33277 wild-type, *P*. *gingivalis* KDP131 (Δ*rgpA*), KDP132 (Δ*rgpB*), KDP129 (Δ*kgp*), KDP133 (Δ*rgpA* Δ*rgpB*), or KDP136 (Δ*kgp* Δ*rgpA* Δ*rgpB*) gingipain-null mutant for 48 h. (D) Primary oral epithelial cells stimulated with 50 μg/ml of *P*. *gingivalis* ATCC 33277 wild-type or KDP136 for 48 h. (E) Ca9-22 cells cultured for 48 h with 50 μg/ml of whole *P*. *gingivalis* W83 cells after incubation with or without at 70°C for 1 h. Total cellular RNA was extracted and transcripts were analyzed by RT-qPCR. Data are representative of three independent experiments, and are shown as means ± SD of triplicate assays. Statistical significant differences are indicated (*, *P*<0.05 compared with respective unstimulated control; §, *P*<0.05 compared with *P*. *gingivalis* alone).

We investigated whether or not gingipain enzyme activities are implicated in the ability of *P*. *gingivalis* to induce IL-33 in Ca9-22 cells and primary oral epithelial cells. The induction of IL-33 mRNA expression by *P*. *gingivalis* W83 whole cells was completely inhibited by Phe-Pro-Arg-chloromethyl ketone (FPR-cmk), an Rgp inhibitor and by KYT-36, a Kgp inhibitor in these cells, respectively (Fig 4A and 4B). The induction of IL-33 mRNA expression in Ca9-22 cells was attenuated by stimulation with *P*. *gingivalis* KDP131 (*ΔrgpA*), KDP129 (Δ*kgp*), KDP133 (*ΔrgpA ΔrgpB*), and KDP136 (*ΔrgpA ΔrgpB* Δ*kgp*) strains, but not with the KDP132 (*ΔrgpB*) strain compared with the *P*. *gingivalis* ATCC 33277 wild-type parent strain (Fig 4C). Therefore, whole *P*. *gingivalis* cells induced IL-33 mRNA expression dependently on RgpA and Kgp. Furthermore, stimulation with *P*. *gingivalis* KDP136 did not induce IL-33 mRNA expression in primary oral epithelial cells (Fig 4D). Incubating *P*. *gingivalis* for 1 h at 70°C to inactivate its enzyme activities completely ablated its ability to induce IL-33 mRNA (Fig 4E). We confirmed that the enzyme activities of Rgps and Kgp were intact in whole *P*. *gingivalis* ATCC 33277 wild-type parent cells, but not in those of comparable mutant *P*. *gingivalis* strains (S3 Fig). These findings indicate that the enzyme activity of gingipains is required for *P*. *gingivalis* to increase IL-33 expression in human gingival/oral epithelial cells.

### Involvement of PAR-2 and PLC in IL-33 induction by gingipains

**Fig 5:**
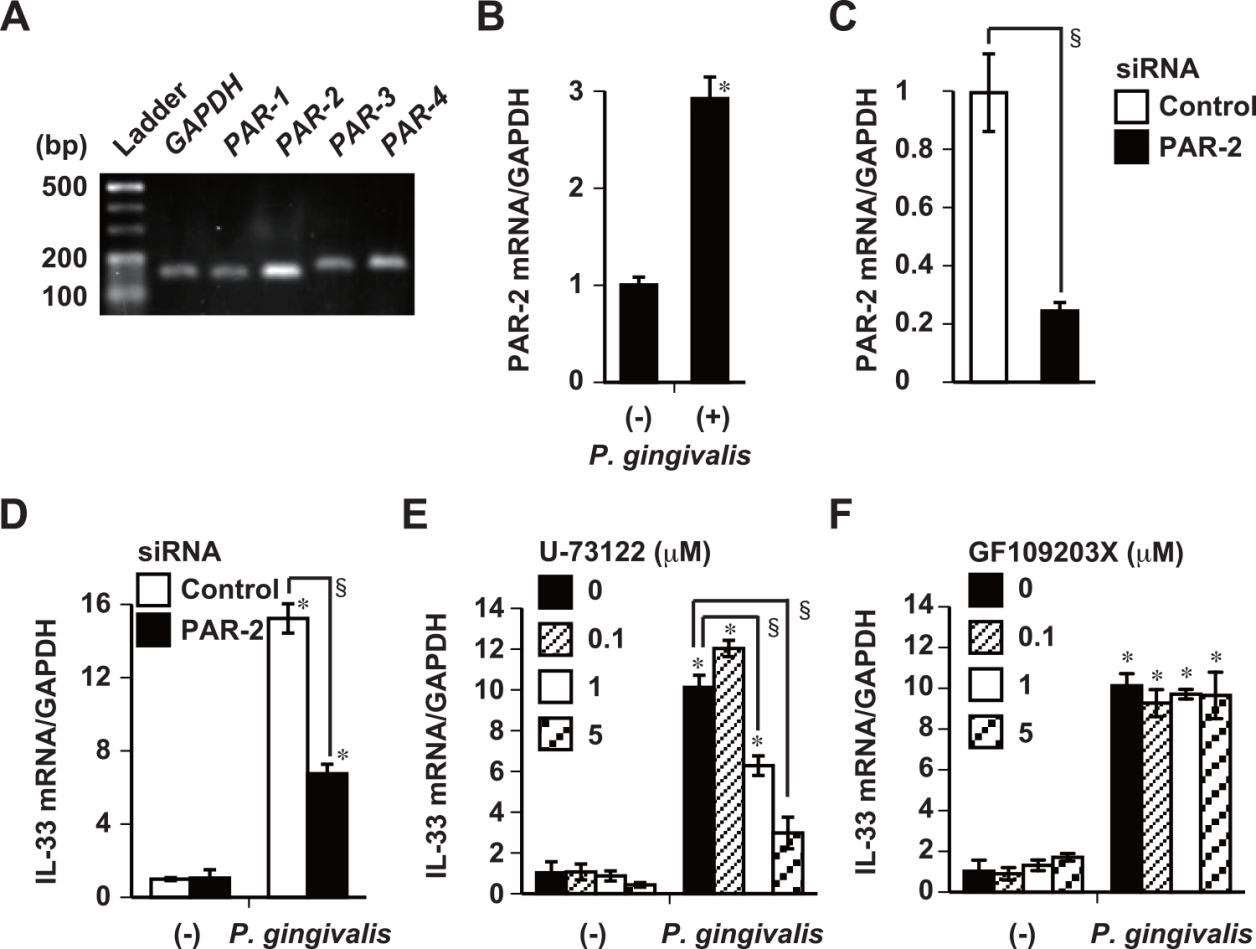
*P*. *gingivalis*-induced IL-33 mRNA expression requires activation of PAR-2 and PLC. (A) Expression of *PAR-1*, *-2*, *-3*, and *-4* mRNA by Ca9-22 cells analyzed by RT-PCR. Data are representative of two independent experiments. Fragments are 156 bp (*GAPDH*), 150 bp (*PAR-1*), 153 bp (*PAR-2*), 170 bp (*PAR-3*), and 177 bp (*PAR-4*), respectively. Ca9-22 cells were transfected with 100 pmol of PAR-2 siRNA or control siRNA for 14 h (C and D), and stimulated with 50 μg/ml of whole *P*. *gingivalis* W83 cells for 48 h (B and D). Ca9-22 cells were incubated with indicated concentrations of U-73122 (PLC inhibitor) (E) or GF109203X (PKC inhibitor) (F) for 30 min, and then stimulated with 50 μg/ml of whole *P*. *gingivalis* W83 cells for 48 h. Expression of PAR-2 mRNA (B and C) and IL-33 mRNA (D, E, and F) was analyzed using RT-qPCR. Data are representative of three independent experiments, and are shown as means ± SD of triplicate assays. Statistical significant differences are indicated (*, *P*<0.05 compared with respective unstimulated control; §, *P*<0.05 compared with *P*. *gingivalis* alone).

Protease-activated receptor belongs to the G protein-coupled family of seven transmembrane domain receptors [38]. Both Rgps and Kgp activate PAR-1 and -2 expressed on human gingival epithelial cells, which produce proinflammatory cytokines [39, 40]. Resting Ca9-22 cells constitutively expressed PAR-1, -2, -3, and -4 mRNA (Fig 5A) and stimulation with whole *P*. *gingivalis* W83 cells increased PAR-2 mRNA expression (Fig 5B). To determine whether or not PAR-2 participates in IL-33 induction, we interfered with PAR-2 mRNA expression in Ca9-22 cells using a PAR-2-specific siRNA. The expression of PAR-2 mRNA was suppressed in 75% of cells transfected with PAR-2 siRNA compared with control siRNA (Fig 5C). The increased expression of IL-33 mRNA induced by whole *P*. *gingivalis* W83 cells was significantly inhibited in cells transfected with PAR-2 siRNA (Fig 5D). Because PAR-2 coupled to G protein is thought to result in the activation of phospholipase C (PLC) and protein kinase C (PKC) [41–43], we investigated whether the PLC pathway is involved in the induction of IL-33 mRNA expression by whole *P*. *gingivalis* in Ca9-22 cells. Incubating Ca9-22 cells with the PLC inhibitor U-73122 (1 μM), significantly inhibited subsequent IL-33 mRNA expression induced by *P*. *gingivalis* (Fig 5E). On the other hand, the PKC inhibitor, GF-109203X, did not inhibit either basal levels of IL-33 or the increase in IL-33 induced by *P*. *gingivalis* (Fig 5F). These findings suggest that the increase in IL-33 expression induced by *P*. *gingivalis* is mediated via the PAR-2-PLC signaling pathway.

### Involvement of p38 and NF-κB in IL-33 induction by gingipains

**Fig 6:**
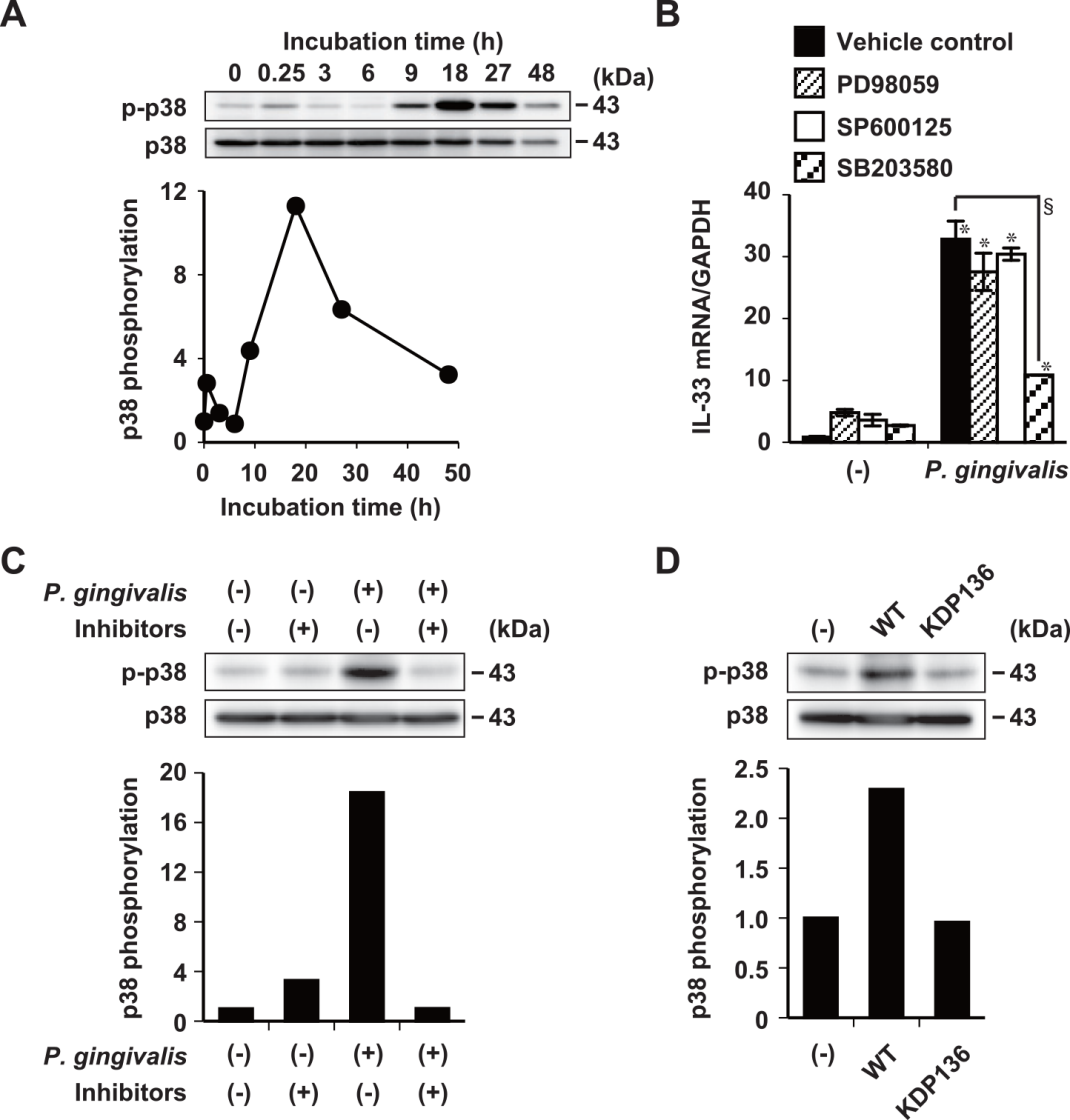
Induction of IL-33 mRNA expression by *P*. *gingivalis* requires activation of the p38 pathway. (A) Ca9-22 cells stimulated for indicated periods with 50 μg/ml of whole *P*. *gingivalis* W83 cells in medium containing 1% FBS. Phosphorylation of p38 was detected in cell lysates by Western blotting against anti-phospho-p38 antibody (p-p38). Controls comprised antibody against total p38. Data are representative of three independent experiments. Relative expression of phosphorylated p38 was quantified using densitometry. Relative expression of phosphorylated p38 was normalized to that of p38. (B) Ca9-22 cells incubated with 10 μM PD98059, SP600125, or SB203580 for 30 min and then stimulated with 50 μg/ml of whole *P*. *gingivalis* W83 cells for 48 h in medium containing 5% FBS. Total cellular RNA was extracted and transcripts were analyzed by RT-qPCR. Data are representative of three independent experiments, and are shown as means ± SD of triplicate assays. Statistical significant differences are indicated (*, *P*<0.05 compared with untreated control; §, *P*<0.05 compared with *P*. *gingivalis* alone). (C) Whole *P*. *gingivalis* W83 cells (50 μg/ml) were incubated with 0.3 μM FPR-cmk plus 0.3 μM KYT-36 for 15 min at 37°C and then used to stimulate Ca9-22 cells for 18 h in medium containing 1% FBS. (D) Ca9-22 cells were stimulated with 50 μg/ml of *P*. *gingivalis* ATCC 33277 wild-type or KDP 136 whole cells for 18 h. Cell lysates were analyzed by Western blotting using anti-phospho-p38 and anti-p38 antibodies. Data are representative of three independent experiments. Relative expression levels of phosphorylated p38 were normalized to levels of p38 and quantified by densitometry. Controls were adjusted to contain 0.1% (v/v) DMSO in media during incubation with inhibitors.

Mitogen-activated protein (MAP) kinase (ERK, JNK, and p38) and NF-κB signaling pathways have been implicated in PAR-2-mediated activation [44, 45]. We therefore assessed whether or not p38 signaling is involved in *P*. *gingivalis*-induced, gingipain-mediated IL-33 regulation in Ca9-22 cells. The phosphorylation of p38 in cell lysates was enhanced after 9 h, and peaked at 18 h of stimulation with whole *P*. *gingivalis* W83 cells (Fig 6A). Incubating cells with the p38 inhibitor SB203580, significantly inhibited the induction of IL-33 mRNA by *P*. *gingivalis* (Fig 6B), whereas PD98059, an ERK1/2 inhibitor, and SP600125, a JNK inhibitor, did not (Fig 6B). Incubating bacterial cells with FPR-cmk plus KYT-36 completely inhibited the subsequent upregulation of p38 phosphorylation by *P*. *gingivalis* W83 (Fig 6C). Stimulation with *P*. *gingivalis* KDP136 did not generate detectable amounts of p38 phosphorylation (Fig 6D).

**Fig 7:**
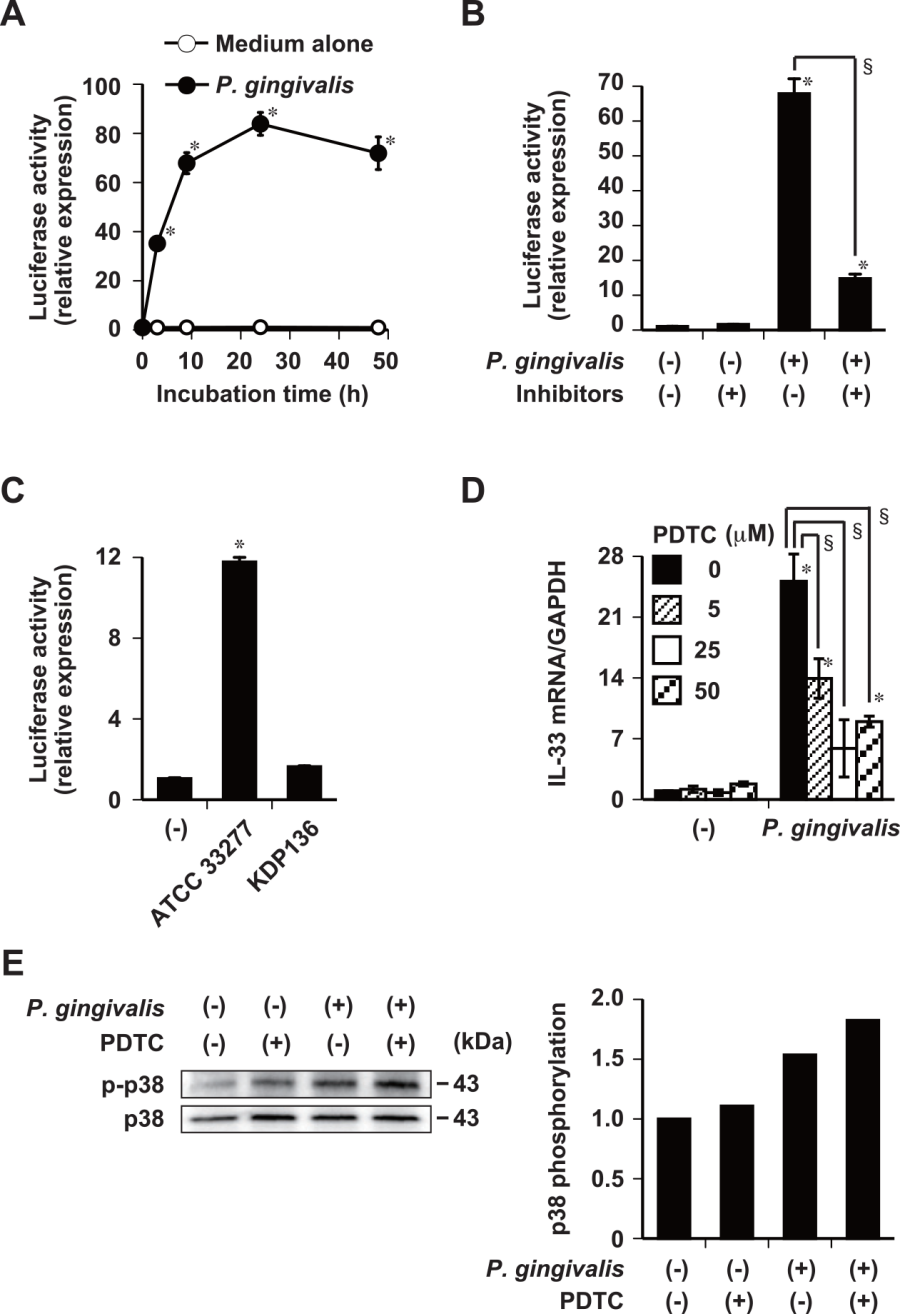
Induction of IL-33 mRNA expression by *P*. *gingivalis* requires activation of the NF-κB pathway. (A) Ca9-22 cells stimulated with 50 μg/ml of whole *P*. *gingivalis* W83 cells for indicated periods. (B) Whole *P*. *gingivalis* W83 cells (50 μg/ml) were incubated with gingipain inhibitors (0.3 μM FPR-cmk plus 0.3 μM KYT-36) for 15 min at 37°C, and then used to stimulate Ca9-22 cells for 9 h. (C) Ca9-22 cells stimulated with 50 μg/ml of whole *P*. *gingivalis* ATCC 33277 wild-type or KDP136 cells for 9 h. (A-C) Cells were transiently transfected with pNFκB-*Metridia* luciferase reporter or control p*Metridia* luciferase reporter plasmids. Amount of secreted luciferase in culture supernatants were analyzed using a luminometer. (D) Ca9-22 cells incubated with indicated concentrations of PDTC (NF-κB inhibitor) for 1 h and then stimulated with 50 μg/ml of whole *P*. *gingivalis* W83 cells for 48 h. Total cellular RNA was extracted and transcripts were analyzed by RT-qPCR. Data are representative of three independent experiments and are shown as means ± SD of triplicate assays. Statistical significant differences are indicated (*, *P*<0.05 compared with respective unstimulated control; §, *P*<0.05 compared with *P*. *gingivalis* alone). (E) Ca9-22 cells were incubated with 25 μM PCTC for 1 h and then stimulated for 18 h with 50 μg/ml of whole *P*. *gingivalis* W83 cells in medium containing 1% FBS. Cell lysates were analyzed by Western blotting against anti-phospho-p38 and anti-p38 antibodies. Data are representative of three independent experiments. Relative expression levels of phosphorylated p38 were normalized to p38 and quantified by densitometry. Control media was adjusted to contain 0.1% (v/v) DMSO during incubation with inhibitor.

We also used luciferase reporter assays to analyze whether or not NF-κB signaling is involved in the induction of IL-33 mRNA expression by *P*. *gingivalis* in Ca9-22 cells that were transiently transfected with a luciferase reporter construct containing the NF-κB response element promoter. Stimulation with *P*. *gingivalis* enhanced NF-κB activity after 3 h and this reached a peak after 24 h. Incubating *P*. *gingivalis* with FPR-cmk plus KYT-36 significantly reduced the subsequent induction of NF-κB activities by *P*. *gingivalis* (Fig 7B) in Ca9-22 cells. Stimulation with *P*. *gingivalis* KDP136 attenuated NF-κB activation in Ca9-22 cells (Fig 7C). Incubating cells with PDTC, an NF-κB inhibitor, significantly inhibited the subsequent induction of IL-33 mRNA expression by *P*. *gingivalis* (Fig 7D), but did not inhibit the phosphorylation of p38 induced by *P*. *gingivalis* at 18 h (Fig 7E). These results suggest that these two signaling pathways work independently of each other in *P*. *gingivalis*-induced IL-33 expression. Collectively, these results indicate that the increase in IL-33 expression induced by *P*. *gingivalis* and mediated by gingipain depends on PAR-2-PLC-p38/NF-κB signaling.

## Discussion

Increasing evidence suggests that epithelium-derived cytokines, including IL-33, IL-25, and TSLP, are critical regulators of Th2 cytokine-mediated inflammation at mucosal sites [3]. The present study found that IL-33 expression was increased in gingival epithelia from patients with chronic periodontitis. We also established that the elevated expression of IL-33 induced by *P*. *gingivalis* was mediated in part via PAR-2 through gingipain-dependent activation in human gingival epithelial cells. Interleukin-33 expression induced by *P*. *gingivalis* was mediated by the PLC, p38, and NF-κB signaling pathways. These results suggest that *P*. *gingivalis*-induced IL-33 expression is associated with the pathogenesis of periodontitis.

Protease-activated receptor-2 is constitutively expressed on gingival epithelial cells from healthy individuals [39] and increased in parallel with the prevalence of *P*. *gingivalis* in gingival crevicular fluid (GCF) from patients with chronic periodontitis [46, 47]. Periodontal treatment decreases PAR-2 expression [46, 48]. Consistent with these facts, PAR-2 expression is increased in response to *P*. *gingivalis* whole cells (Fig 5B) and culture supernatants [49] in human gingival epithelial cells. *P*. *gingivalis*-induced IL-33 expression was attenuated in PAR-2 knockdown Ca9-22 cells (Fig 5D). Cellular activation is mediated by PAR-2 binding to ligand after the receptor is activated by proteolysis, or to tethered ligand peptide agonists in a proteolysis-independent manner [38]. We determined that IL-33 expression needed to be increased by *P*. *gingivalis* for the enzymatic activation of gingipains, because heating the bacteria to inactivate the enzymatic activities of gingipains abolished the subsequent *P*. *gingivalis*-mediated increase in IL-33 mRNA (Fig 4E). The expression of TSLP or IL-25 is induced in bronchial epithelial cells via PAR-2 by serine proteases from airborne pathogens, such as *Alternaria* or house dust mites [50, 51]. We confirmed that TSLP and IL-25 mRNA expression was obviously and gingipain-dependently increased by fresh *P*. *gingivalis* cultures (S1A and S1B Fig) and by whole *P*. *gingivalis* cells (S1C and S4 Figs), similar to that of IL-33 mRNA expression in Ca9-22 cells. The production of Th2 cytokines and chemokines from human mast cells in response to IL-33 is enhanced by adding TSLP [52]. Cross-regulation among IL-33, IL-25, and TSLP might play important roles in the persistence of Th2 cytokine-mediated inflammation. The activation of PAR-2 induces canonical G protein and β-arrestin-dependent signaling followed by the translocation of β-arrestin-1 from the cytosol to the plasma membrane and the subsequent endocytosis of PAR-2-β-arrestin-1 complexes in early endosomes [38, 53]. However, the internalization of phosphorylated-PAR-2 is through a canonical dynamin-, clathrin-, and β-arrestin-dependent pathway in this context [54]. The expression of IL-33 mRNA induced by *P*. *gingivalis* was partly attenuated in β-arrestin-1 knockdown Ca9-22 cells compared with control cells (S5 Fig). These results suggest that activated-PAR-2 internalization and the formation of PAR-2-β-arrestin-1 complexes are required for *P*. *gingivalis*-induced IL-33 induction. However, the processes of *P*. *gingivalis*-induced β-arrestin-1 mobilization and association with *P*. *gingivalis* in Ca9-22 cells remain unclear. Lamont *et al*. (1995) reported that *P*. *gingivalis* invasion of oral epithelial cells is inhibited in the presence of cytochalasin D which also inhibits actin polymerization, or nocodazole which depolymerizes microtubules [55]. The present study found that blocking *P*. *gingivalis* invasion using cytochalasin D or nocodazole impaired *P*. *gingivalis*-induced IL-33 expression (Fig 2F and 2G). The above and the present findings suggest that *P*. *gingivalis* invasion is necessary to increase IL-33 expression. Others have shown that *P*. *gingivalis* invades gingival epithelial cells via endocytosis, which is mediated by the binding of RgpA adhesin peptide A44 to host cells [56]. Both RgpA and Kgp have hemagglutinin/adhesin domains that interact with fibrinogen, fibronectin and laminin, whereas RgpB does not [57]. The up-regulation of IL-33 by *P*. *gingivalis* was not attenuated by stimulation with *P*. *gingivalis* KDP132 (Δ*rgpB*), which lacks hemagglutinin/adhesin domains (Fig 4C). Gingipains are notably essential for the maturation of *P*. *gingivalis* fimbriae [58], which are required for *P*. *gingivalis* attachment [59] and internalization into epithelial cells [60]. However, it is unknown whether purified gingipains induce IL-33 production in human gingival epithelial cells. Further experiments are needed to understand this. These findings, in conjunction with our results, suggest that both *P*. *gingivalis*-induced PAR-2 activation and the endocytosis of *P*. *gingivalis* conceivably participate in the increased expression of IL-33 in gingival epithelial cells.

We detected p38 phosphorylation in Ca9-22 cells at 18 h after stimulation with whole *P*. *gingivalis* cells (Fig 6A). The upregulation of IL-33 mRNA by whole *P*. *gingivalis* cells was not mediated via either the ERK or the JNK pathways (Fig 6B). One study found that ERK and JNK became phosphorylated in human gingival epithelial cells at 5 min after infection with *P*. *gingivalis* whereas p38 did not [61]. In contrast, CCL20 mRNA that is expressed in human gingival epithelial cells at 16 h after infection with *P*. *gingivalis* is blocked by inhibiting p38, but not JNK [62]. This suggests that p38 activation is involved in the late-phase response to IL-33 regulation. We clarified that the increased expression of IL-33 was induced by *P*. *gingivalis* through gingipains via the PAR-2, PLC, p38, and NF-κB signaling pathways.

The expression of IL-33 is increased in response to proteases, pathogen-associated molecular patterns (PAMPs), and proinflammatory stimuli [1, 6, 63, 64]. *P*. *gingivalis* possesses various virulence factors, such as LPS (TLR2 and TLR4 ligand) [33], fimbriae (TLR2 ligand) [31], and lipoprotein (TLR2/1 ligand) [35]. The present study found that fimbriae and PGTP2-RL, a lipopeptide from *P*. *gingivalis*, were completely inactive, whereas *P*. *gingivalis* LPS was only weakly active in human gingival epithelial cells (Fig 2D), due to low levels of TLR2 expression in these cells [65]. Nile *et al*. reported that *P*. *gingivalis* LPS (TLR2 and TLR4 ligand) induces IL-33 expression in human monocytes [6]. We also determined that IL-33 expression is increased by fimbriae or PGTP2-RL in bone marrow-derived dendritic cells from WT mice, but not from TLR2-deficient mice (unpublished data).

Full-length IL-33 is released from necrotic cells and subsequently acts as an alarmin [6, 66, 67, 68]. Stimulation with extracellular ATP or mechanical stress without cell death also results in IL-33 production [36, 37]. Levels of IL-33 in GCF do not differ between healthy donors and patients with chronic periodontitis [69] and IL-33 is not a component of GCF in inflamed periodontal tissues from patients with chronic periodontitis [70]. Indeed, our ELISA findings indicated very low levels of IL-33 (25 pg/ml) in supernatants from Ca9-22 cells stimulated with 50 μg/ml of *P*. *gingivalis* W83 for 4 d (data not shown). Interleukin-33 localized in the nucleus of resting Ca9-22 cells (Fig 3B and S2 Fig), whereas IL-33 induced by *P*. *gingivalis* re-localized in the cytoplasm of Ca9-22 cells (Fig 3B). The endogenous full-length IL-33 (30 kDa) induced by *P*. *gingivalis* in the total cell lysate appeared to have biological activity (Fig 3A). When Ca9-22 cells were incubated for up to 4 d with 50 μg/ml of whole *P*. *gingivalis* W83 cells, lactate dehydrogenase (an indicator of necrosis) was detected in the culture supernatants at 2 d (S6B Fig). These results suggest that a small amount of *P*. *gingivalis*-induced IL-33 is released from the cells after necrosis. Cell viability was slightly reduced in the cells stimulated with *P*. *gingivalis* at 1 to 2 d after incubation, but not at 4 d after incubation (S6A Fig). Gingipains can directly cleave cytokines such as TNF-α [71] and IL-8 [26], and induce caspase-3-dependent apoptosis in human gingival epithelial cells [72]. Caspase-3 proteolytically processes IL-33 to the inactive form after apoptotic cell death [66, 67]. Under these experimental conditions, IL-33 might be directly cleaved by gingipains or inactivated by caspase-3. However, whether or not the action of released IL-33 as an alarmin in periodontal tissues is gingipain concentration-dependent remains unknown. The turnover rate of gingival epithelial cells is 4–6 days [73], which is high enough to allow rapid replacement of the cells by microbial challenge. Alternatively, IL-33 acts as a dual-functional molecule that works as an endogenous nuclear protein [74]. Ali *et al*. reported that full-length IL-33, which localized in both the cytosol and nucleus, interacts with NF-κB p65 and impairs p65-mediated transactivation [75]. Thus the accumulation of intracellular IL-33 induced by *P*. *gingivalis* might attenuate the p65-mediated proinflammatory reaction in gingival epithelial cells. The function of IL-33 induced by *P*. *gingivalis* in epithelial cells might primarily be intracellular, and secondarily as an alarmin upon release into the extracellular milieu. The roles of IL-33 derived from gingival epithelial cells, which could include the modulation of innate immune function, need to be defined by further studies and the implications for the pathogenesis of periodontal diseases need to be assessed. Infection with *P*. *gingivalis* appears to promote the PAR-2/IL-33 axis through a gingipain-dependent mechanism in human gingival epithelial cells.

## Supporting Information

S1 Fig *P*. *gingivalis* increases IL-25 and TSLP mRNA expressions in gingival epithelial cells. Ca9-22 cells were infected with fresh *P*. *gingivalis* W83 cultures at an MOI of 0.5 for the indicated periods (A), or at the indicated MOI for 48 h (B). (C) Ca9-22 cells were stimulated for 48 h with indicated concentrations of whole *P*. *gingivalis* W83 cells. Total cellular RNA was extracted and transcripts of IL-25 and TSLP were analyzed by RT-qPCR. Data are representative of three independent experiments, and are shown as means ± SD of triplicate assays. Statistical significant differences are indicated (*, *P*<0.05 compared with untreated control).
(EPS)

S2 Fig Nuclear expression of IL-33 in resting gingival epithelial cells. Cell lysates, cytoplasmic extracts or nuclear extracts from Ca9-22 cells were analyzed by Western blotting with anti-human IL-33 mAb or anti-human GAPDH mAb. Data are representative of three independent experiments.
(EPS)

S3 Fig Amidolytic activities of various gingipain-mutant strains of whole *P*. *gingivalis* cells. Amidolytic activities of *P*. *gingivalis* whole cells were assayed at 37°C with 0.5 mM BA-pNA (A) or 0.5 mM HEK-MCA (B) for 1 h. Results are expressed as absorbance of *p*-nitroaniline (A) or 7-amino-4-methylcoumarin (B) released from substrate, respectively.
(EPS)

S4 Fig Involvement of gingipains in *P*. *gingivalis*-induced IL-25 and TSLP mRNA expression in gingival epithelial cells. (A and B) Whole *P*. *gingivalis* W83 cells (50 μg/ml) were incubated with 0.3 μM FPR-cmK or 0.3 μM KYT-36 for 15 min at 37°C. Ca9-22 cells (A) or primary oral epithelial cells (B) were stimulated with the treated or untreated *P*. *gingivalis* whole cells for 48 h. Ca9-22 (C) or primary oral epithelial (D) cells were stimulated with 50 μg/ml of *P*. *gingivalis* ATCC 33277 wild-type or KDP136 for 48 h. Total cellular RNA was extracted and IL-25 and TSLP transcripts were analyzed by RT-qPCR. Data are representative of three independent experiments and are shown as means ± SD of triplicate assays. Statistical significant differences are indicated (*, *P*<0.05 compared with respective unstimulated control; §, *P*<0.05 compared with *P*. *gingivalis* alone).
(EPS)

S5 Fig *P*. *gingivalis*-induced IL-33 mRNA expression partially requires expression of β-arrestin-1. Ca9-22 cells were transfected with 100 pmol of β-arrestin-1 siRNA or control siRNA for 12 h, and cells were stimulated with 50 μg/ml of whole *P*. *gingivalis* W83 cells for 48 h. The expression of IL-33 mRNA was analyzed by RT-qPCR. Data are representative of three independent experiments, and are shown as means ± SD of triplicate assays. Statistical significant differences are indicated (*, *P*<0.05 compared with respective unstimulated control; §, *P*<0.05 compared with negative control siRNA).
(EPS)

S6 Fig Cell viability and cytotoxicity of gingival epithelial cells after stimulation with *P*. *gingivalis*. Ca9-22 cells were stimulated with 50 μg/ml of whole *P*. *gingivalis* W83 cells for the indicated periods, and then the luminescence of NanoLuc substrate (A) or the absorbance of LDH (B) was measured using a luminometer or spectrophotometer, respectively. Data are representative of three independent experiments and are shown as means ± SD of triplicate assays.
(EPS)
